# Epidemiological and genomic analysis of *Acinetobacter baumannii* strains from retailed raw meat

**DOI:** 10.1016/j.heliyon.2024.e41487

**Published:** 2024-12-25

**Authors:** Leila Hamze, Raquel Garcia-Fierro, Antoine Drapeau, Pauline François, Andrea Endimiani, Jean-Yves Madec, Marisa Haenni, Vincent Perreten, Agnese Lupo

**Affiliations:** a ANSES - Université de Lyon, Unité Antibiorésistance et Virulence Bactériennes, Lyon, France; b Institute for Infectious Diseases (IFIK), University of Bern, Bern, Switzerland; c Division of Molecular Bacterial Epidemiology and Infectious Diseases, Institute of Veterinary Bacteriology, Vetsuisse Faculty, University of Bern, Bern, Switzerland

**Keywords:** International clone 11, Clonal complex 33, *bla*_OXA-23_, Transformation, Carbapenem-resistance, Food

## Abstract

*Acinetobacter baumannii* causes hospital-acquired infections in human patients with compromised immune system. Strains associated to nosocomial infections are often resistant to carbapenems and belong to few international clones (IC1-11). *A*. *baumannii* strains have been found in extra-hospital sources including food products. While molecular epidemiology of *A*. *baumannii* is well described in hospital settings, extra-hospital settings remain poorly investigated.

In the frame of two screening campaigns for the presence of Gram-negative bacteria in retailed raw meat, we collected 70 *A. baumannii* isolates. To investigate if there was a genetic link between food isolates and those causing infections in humans, a core-genome pyMLST analysis was conducted including genomes from different sources as well as representatives of the IC1-11 (n = 224) retrieved from the NCBI database.

Strains from raw meat were genetically diverse with 49 sequence types present among the 70 isolates. The core-genome phylogenetic analysis demonstrated that some *A*. *baumannii* strains from raw meat shared high genomic similarity with strains associated to human infections carrying carbapenem-resistance genes and belonging to IC11 and other clonal complexes (CC) that are emerging globally, like CC33. Strains from raw meat were able to acquire genes conferring carbapenem-resistance *in vitro*.

If *A*. *baumannii* cannot be considered as a food-borne pathogen, colonization of raw meat can favor the propagation of this species in the community, facilitating the entrance of novel clones in the hospital environment. Once entering hospital settings, susceptible clones could turn into multidrug-resistant lineages under strong selective pressure. To avoid this risk, accurate hands and kitchen utensils hygiene should be recommended to all those in contact with raw meat.

## Highlights

• *A. baumannii* strains from retailed raw meat are genetically diverse among them.
• Some strains are genetically similar to those associated to infections in humans.
• *A. baumannii* from raw meat are able to acquire carbapenem-resistance *in vitro*.

## 1 Introduction

*Acinetobacter baumannii* is infamous for hospital-acquired infections in human patients with compromised immune system [1,2]. *A*. *baumannii* strains causing nosocomial infections and outbreaks are multidrug-resistant (MDR). Of particular concern is the ability of this species to acquire genetic determinants, which confer resistance to carbapenems [3] and even to the most recently developed cefiderocol [[4], [5], [6]]. Among the genetic determinants conferring carbapenem-resistance, *bla*_OXA-23_ gene is the most prevalent, usually carried by Tn*2006* [7]. The gene *bla*_NDM-1_ embedded in Tn*125* transposon is emerging [8].

The ability of *A*. *baumannii* strains to survive on surfaces of hospital items and colonize hands of medical caregivers favor the propagation of this opportunistic pathogen in the hospital settings, generating outbreaks and epidemics [9]. Strains able to generate nosocomial outbreaks belong to few international clones (IC). So far, eleven (IC1-11) of these globally disseminated lineages have been characterized [[10], [11], [12]]. While molecular epidemiology of *A*. *baumannii* is well described in human hospital settings, with IC2 as the most common lineage causing outbreaks and epidemics [12], the epidemiology of this species in extra-hospital sources remains poorly investigated. In particular, the epidemiological connections between hospital and extra-hospital settings are debated, with reports suggesting that *A*. *baumannii* is a strictly nosocomial pathogen [13] and more recent investigations pointing to *A*. *baumannii* as a One Health issue [14]. Actually, *A*. *baumannii* strains have been found in companion animals [[15], [16], [17]] and wildlife [18], in food producing animals [[19], [20], [21]], in food products like vegetables [22] and tap water [23], and frequently in retailed raw meat [[24], [25], [26]]. Typing of isolates from food sources with standardized comparable methods is rare [21,25], whereas analyses based on whole-genome sequencing are inexistent. The contribution of *A*. *baumannii* in food products to the hospital epidemiology and antibiotic resistance issue remains unclear.

In the current study, *A*. *baumannii* isolates recovered from retailed raw meat were analyzed by whole-genome-sequencing, together with genomes of strains associated to infections in humans and other sources in order to understand their phylogenetic relationships. Genomes representative of IC1-11 were included, as well. Furthermore, the potential of *A*. *baumannii* strains from raw meat to acquire resistance to antibiotics used in human medicine was investigated *in vitro*.

## 2 Material and methods

### 2.1 Isolates collection

Isolates were collected in the frame of two surveillance campaigns for the presence of Gram-negative bacteria in retailed raw meat in Switzerland (November 2012–May 2013) and in France (December 2018–July 2019).

For the first campaign, the methodology of strains isolation was reported previously [25]. In the frame of the second campaign, 42 chicken meat packages were acquired from groceries in Lyon, France. The samples were handled in the laboratory ANSES-Lyon, in sterile atmosphere, and isolates were obtained on CHROMAgar Acinetobacter plates (MAST diagnostics, France) as described previously [27]. From each plate, one red colony per morphological type was stored for further characterization.

### 2.2 Antibiotic susceptibility testing

Susceptibility to a panel of 12 antibiotics (Table S1) was evaluated by disc diffusion, for eleven antibiotics, according to Antibiogram Committee of the French Society for Microbiology (CA-SFM; https://www.sfm-microbiologie.org/). Susceptibility to colistin was determined by broth microdilution according to EUCAST recommendations (https://www.eucast.org/eucastguidancedocuments). The strains *Escherichia coli* ATCC 25922 and *Pseudomonas aeruginosa* ATCC 27853 were used as quality controls.

### 2.3 Genome sequencing and assembly

From each isolate, DNA was extracted using a NucleoSpin Microbial DNA extraction kit (Macherey-Nagel, Hoerdt, France). Libraries synthesis and genomes sequencing were outsourced to an external service generating 2x150 paired-end reads (Eurofins Genomics, Germany). Reads quality was determined (FastQC v. 0.11.9) and *de novo* assembly obtained using Shovill v.1.0.4. Reads and assemblies are available from PRJNA1076118 bio project.

### 2.4 Bioinformatics analysis

Presence of resistance genes was investigated using StarAMR (v.0.9.1). Sequence types (STs) were assigned according to the Institute Pasteur scheme (PubMLST accessed on December 21, 2023) [28] and clustering in clonal complexes (CC) or IC was evaluated by eBurst [29].

The NCBI RefSeq *A*. *baumannii* genomes repertoire (n = 8129, accessed on August 31, 2023) contained 141 genomes with the same ST or single locus variant (SLV) of isolates collected from raw meat. Genomes (n = 83) representative of ICs [16,30,31] were included as well. For all 294 genomes (70 from raw meat, 83 ICs representative, 141 SLV of isolates from raw meat), the quality of the assembly was verified (Quast v.5.0.2) and only genomes presenting less than 500 contigs were retained for further analysis. Identification was confirmed by ANI determination (fastani v.1.34). Level of contamination was evaluated using kraken 2 (v. 2.1.2), whereas completeness of the genomes was evaluated using Busco v. 5.7.1. The 294 genomes were included in a pyMLST (v.2.1.5) analysis using the pre-defined *A*. *baumannii* core-genome (2390 alleles) [11]. The phylogenetic tree mid-point rooted was visualized and edited with iTOL v.6. After annotation (Bakta v.1.9.3), presence/absence of genes in the pangenome was analyzed using PPanGGOLiN (v.2.1.0).

### 2.5 Horizontal genes transfer assays

Isolates from raw meat were used as recipient in assays of natural transformation [32] using as donor the DNA extracts of strain #38208 (GCF_030377345.1) and #51877 (GCF_030377475.1), both carriers of *bla*_OXA-23_ gene on Tn*2006* [16].

## 3 Results

### 3.1 Collection of isolates and genomes sequence type assignment

Isolates from the first campaign (n = 32) were found in different meat samples, including chicken, turkey, veal, pork and beef. In the second campaign, 21 out of 42 chicken meat samples tested positive for *A*. *baumannii* and 38 different isolates were collected (Table S1).

Forty-nine STs were assigned to the 70 isolates, with five *A*. *baumannii* isolates from Switzerland belonging to ST353. ST109, ST348, ST946, and ST1890 were assigned each to three different isolates. The remaining STs were sporadic. Some isolates were assigned to SLV of STs assigned in the previous analysis [25] (Table S2). These changes were probably due to the novel sequencing methodology. Besides, repeated cultivation passages of the strains could have led to point mutations.

Thirteen chicken samples carried multiple *A*. *baumannii* isolates belonging to different STs. Few samples (W2SB and W8SA) carried three different clones, and one (W7SC) carried up to four clones. Meat samples packaged in the same meat processing site carried similar clones. For instance, ST348 isolates (n = 3) and ST349 (n = 2) were all from processing site B-CH, ST353 (n = 5) from site A-CH, ST357 isolates (n = 2) and ST358 (n = 2) were from site C-CH, and ST1890 isolates (n = 2) from site E-FR. On the contrary, the same ST was found in samples processed in different sampling sites, for example ST946 was found in I-FR and E-FR, ST106 in C-FR and I-FR, and ST1337 isolates were found in sites E-FR and L-FR (Table S1). ST109 was assigned to isolates from Switzerland (#53407) and from France (#54660 and #54721). Similarly, ST42 and ST364 were assigned each to isolates collected in Switzerland (ST42: #53392; ST364: #53378) and in France (ST42: #54716; ST364: #54687).

To analyze the genomic relationships between isolates from raw meat (n = 70) and those from other sources, genomes from the RefSeq NCBI database displaying the same ST or belonging to SLV (n = 141) of those of isolates from raw meat were uploaded, together with representative of IC1-11 (n = 83) (Table S1). For eleven out of those 294 genomes the source was not specified, otherwise genomes belonged mostly to isolates associated to human infections (n = 193), followed by inanimate surfaces (hospital, n = 5; school, n = 1), animals (n = 9), the environment (n = 3), and waste water (n = 2). The geographical origin was highly diverse, including Asia (n = 89), North America (n = 43), Europe (n = 114), Central America (n = 5), Africa (n = 9) and Oceania (n = 4). For 14 genomes, geographical origin was not reported. The 294 genomes were from isolates collected during 1992–2022 (Table S1).

### 3.2 Antibiotic susceptibility and acquired resistance genes

The 70 isolates from raw meat displayed susceptibility to the 12 tested antibiotics (eleven tested by disk diffusion and colistin tested by MIC determination), with the exception of 12 isolates that were resistant to tetracyclines and carried the *tet*(39) gene, one (#53409) that was ciprofloxacin resistant presenting *gyrA* and *parC* polymorphism, and two isolates that were colistin-resistant (MIC: 6–8 mg/L) with one of those (#53392) presenting mutations in *pmrA* and *pmrB* loci (90 % nucleotide identity with reference strain K09_14 (Accession number ASM863263_v1) [33] (Table S1).

**Fig. 1:**
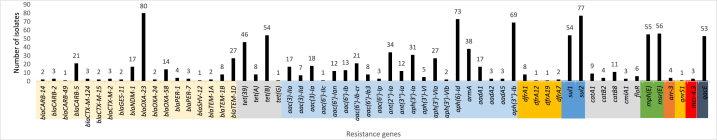
Occurrence of genes conferring antibiotic resistance in 294 *Acinetobacter baumannii* genomes.

Antibiotic resistance genes were searched also in the 224 genomes from NCBI. The gene *bla*_OXA-23_ was the most frequent among carbapenemase-encoding genes (n = 80/294 genomes), followed by *bla*_NDM-1_ (n = 17/294) and *bla*_OXA-58_ (n = 14/294). Other frequent resistance genes were *aph*(3”)-*Ib* (n = 69) and *aph*(6’)-*Id* (n = 73), present also in isolates from raw meat (#54696 and #54725). Polymorphisms in genes *gyrA* and *parC* conferring resistance to fluoroquinolones were common, similarly to the *tet*(39) gene (n = 46/294) (Fig. 1).

### 3.3 pyMLST and eBurst analysis

**Fig. 2:**
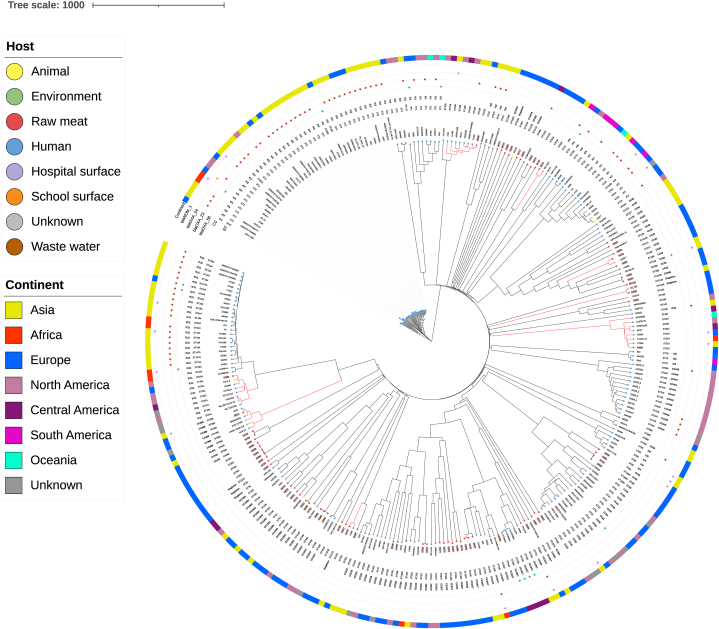
Phylogenetic tree of 294 *Acinetobacter baumannii* isolates from raw meat and genomes retrieved from RefSeq NCBI repository. The allelic differential matrix was constructed on 2390 alleles using pyMLST. The representation was obtained using iTOL v.6. Branches in red highlight isolates from raw meat and other sources sharing genomic similarities. Label of isolates from raw meat are highlighted in pink. CC: clonal complex; ST: sequence type.

The core-genome phylogenetic analysis based on 2390 alleles and including 294 *A*. *baumannii* (224 from NCBI and 70 from raw meat) generated a tree split into two major clades, one including only IC2 isolates and the other one including the remaining isolates. Isolates from raw meat presented high genetic diversity and were distinct from ICs representative genomes, with the exception of genomes belonging to IC11 (Fig. 2). On the contrary, genomes belonging to a given IC clustered together (Fig. 2).

Besides such general diversity, phylogenetic proximity could be observed between isolates from raw meat and those from other sources. For instance, #53410 (Switzerland, pork meat) was close (39 allelic differences) to isolate cpe017 isolated from hospital surfaces in United Kingdom. Similarly, isolate #54680 (France, chicken meat) was close to AB_353 isolated from hospital surfaces in Asia (25 allelic differences). Isolate #54688 (France, chicken meat) was close to a group of isolates associated to human infections occurring in Central and North America and Asia (35–136 allelic differences, Fig. 2). Isolate #54665 (France, chicken meat) was close to isolates associated to different types of infections in human patients from North and Central America, Oceania and Europe but also to isolates from surfaces of a school in Ghana (Fig. 2). Isolate #54722 was close to A158 found in the bile of a human patient in Asia (65 allelic differences). Isolate #53411 (Switzerland, chicken meat) displayed genomic similarities with isolates 3159 and MRSN32875 associated to a wound and a respiratory infection in human patients from China and Germany, respectively (19–45 allelic differences). In turn, these isolates were close to isolates from water sources and animals (Fig. 2). Isolate #54683 was close to isolates C3T1-2 and 118362 isolated from a cow and a perirectal swab of a human patient, respectively, both from North America (Fig. 2, Table S2).

Some isolates from human sources sharing genomic similarity with isolates from raw meat carried a carbapenemase encoding gene (Fig. 2, Table S1).

**Fig. 3:**
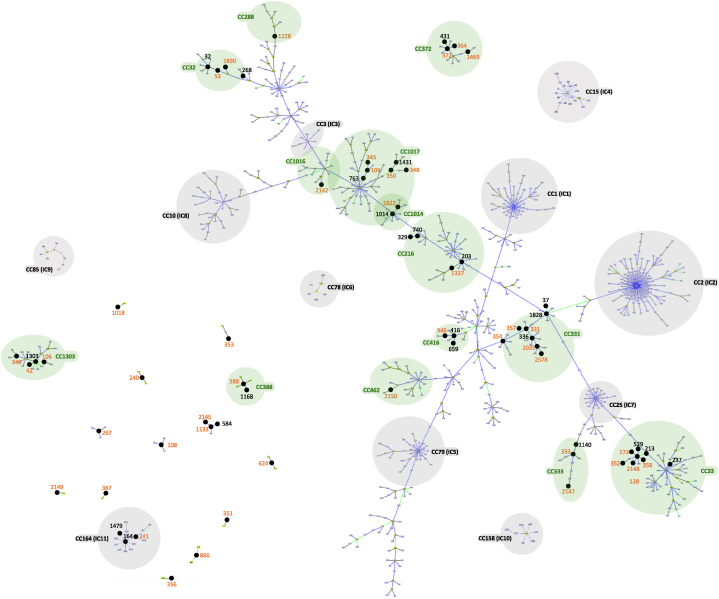
Representation of eBurst analysis of sequence types attributed to *Acinetobacter baumannii* isolates from raw meat (in orange) and those retrieved from RefSeq NCBI sharing the same sequence type or single locus variants (in black). Clonal complexes are highlighted in green and previously described international clones in gray.

Isolates from raw meat sharing genomic proximity with isolates from other sources were assigned to different STs in turn belonging to several CCs. CC372, here defined for the first time, contained 13 STs associated to isolates from humans from all over the globe (Russia, Japan, Thailand, and United States) (Fig. 3). An additional large CC was CC1017 [34] containing ST109, ST345, ST763, ST350 and ST1431. In CC1017 genomes assigned to ST109 segregated in two groups, one of these included isolates from patients attending the same hospital in Central America, suggesting the occurrence of an outbreak [35]. CC33 [21] included several isolates from raw meat, likewise CC1303 [36] (ST349, ST1303, ST42, and ST106). Furthermore, one isolate from chicken meat (#54683) was assigned to ST241 that, together with ST164 and ST1479, belonged to the CC164 recently defined as IC11 (Fig. 3). Genomes belonging to IC11 constituted the largest node containing similar genomes, predominantly from human patients (33) from Thailand, suggesting the occurrence of an outbreak. Furthermore, most of IC11 isolates (21/33) carried a *bla*_OXA-23_ gene, whereas a smaller proportion (3/33) carried the carbapenemase-encoding gene *bla*_OXA-58._ Three out of 33 IC11 genomes the metallo-carbapenemase encoding gene *bla*_NDM-1_ (Table S1).

To investigate if the biological source or the geographical origin of the isolates was governed by the acquisition of accessory genes, a presence/absence analysis of genes in the pangenome was carried out. Besides, a hierarchical clustering governed by these parameters was not observed (data not shown).

### 3.4 Transformation assays

Five isolates (#53390 and #53412, ST349; #54680, ST2146; #54698, ST2148; #54711, ST624) generated transformants with DNA from strain #38208 carrying the *bla*_OXA-23_ gene. One single isolate (#54693, ST1133) generated transformants with DNA from strain #51877. All transformants displayed resistance to meropenem and imipenem (MIC >32 mg/L).

## 4 Discussion

*A. baumannii* isolates were found in 25 % of Swiss samples from 2013 to 2014 and 50 % of French samples from 2018 to 2019, respectively. This different prevalence could reflect several differing factors between the two campaigns, linked to the methodology of screening (an enrichment step was performed in the frame of the screening conducted in France, and two different selective media were used), to the period of the analysis and to the origin of the meat. Albeit such differences, the occurrence of *A*. *baumannii* isolates was high in both screenings, similarly to the reports from Australia (March–June 2014) in raw chicken meat and pork (59/164, 36 % positive samples) [26] and Iran (20 %, period 2017–2020) [37,38]. Lower prevalence was reported from Portugal in different types of meat (7/50 samples, 14 %; period 2013–2014) [24] and Lebanon in beef samples (4/50 samples, 8 %; period 2012–2013) [21].

Carvalheira et al. observed that one raw meat sample could carry several *Acinetobacter* spp. [24]. We observed that most samples carried several different *A*. *baumannii* clones. In general, the 70 raw meat isolates displayed genetic diversity and were assigned to 49 different STs. Furthermore, *A. baumannii* isolates collected from raw meat presented susceptibility to antibiotics used in human medicine, in agreement with previous observations reporting susceptibility to carbapenems [21,24,26], with few exceptions [37,38]. In the isolates of the current study, the most frequent resistance was towards tetracyclines in isolates carrying *tet*(39) gene. Recently, Savin et al. reported that this gene was mostly found in *A*. *baumannii* isolates from poultry environment [39]. Tetracyclines are allowed antibiotics for the treatment of infections in poultry in several countries [40], potentially favoring the selection of this resistance gene. We might thus speculate that *A*. *baumannii* isolates from raw meat originated from animals. However, it appears that the presence of *A*. *baumannii* in poultry occurs at low prevalence and mostly in young birds [41]. Low prevalence and high diversity have been reported also from cows [19,21] and from pigs, where isolates are distinct from international lineages [20]. Intriguingly, some *A*. *baumannii* clones (ST42, ST109, ST351, and ST364) occurred in the two sampling campaigns, despite the lapse of time and the geographical difference between the two campaigns. Furthermore, some clones were found in meat samples from the same meat-processing site, suggesting persistence in this environment. Colonization of meat samples from hands of operators in meat-processing sites cannot be excluded, similarly to what is observed in hospital settings, where hands colonization of hospital personnel is responsible for the propagation of this opportunistic pathogen [9]. *A*. *baumannii* colonization of hands of healthy humans is largely unknown.

Isolates from raw meat were diverse but some belonged to international lineages (IC11), other to large CCs undergoing global dissemination (CC372, CC1017, CC33) or less expanded CCs like CC216 or CC32. These CCs included isolates associated exclusively to human patients like IC11 [10], to humans and animals like CC1017 (PubMLST database), CC1303 and CC33 [21], to food CC216 [21], to humans and the environment CC32 [42]. The core-genome phylogenetic analysis permitted to highlight that some isolates from raw meat shared high genomic similarities with isolates originating from hospital settings. While isolates from raw meat were susceptible to antibiotics used in human medicine, close isolates from hospital settings carried carbapenemase-encoding genes. However, isolates from raw meat belonging to several STs demonstrated the ability to transform *in vitro* and acquire the *bla*_OXA-23_ gene, suggesting that raw meat isolates might acquire resistance once colonizing humans and undergoing selective pressure.

## 5 Conclusion

*A. baumannii* is largely present in raw meat, although with varying prevalence according to the type of meat, geographical region and period. Despite the genetic similarities observed between the *A*. *baumannii* strains found in raw meat and those in clinical environments, there is no evidence establishing a direct connection between the two populations. Whether the occurrence of *Acinetobacter* in both environments is coincidental or whether there is a missing link between both contexts remains to be documented. *A*. *baumannii* presence on raw meat is not menacing human health directly, like a food-borne pathogen. Nevertheless, this colonization can favor the propagation of this opportunistic pathogen in the community, thus facilitating the entrance of novel clones in hospital settings, which could turn into multidrug-resistant lineages under strong selective pressure. To avoid this risk, strict hands and kitchen utensils hygiene should be recommended to all those in contact with raw meat. The source of *A*. *baumannii* colonization of raw meat remains unknown. Consequently, in order to understand the One Health dimension of this opportunistic pathogen, it would be necessary to consider the spill over from humans and increase knowledge of *A*. *baumannii* colonization of healthy individuals.

## CRediT authorship contribution statement

**Leila Hamze:** Writing – original draft, Methodology, Investigation, Formal analysis. **Raquel Garcia-Fierro:** Investigation, Formal analysis. **Antoine Drapeau:** Software, Formal analysis. **Pauline François:** Methodology, Formal analysis, Data curation. **Andrea Endimiani:** Writing – review & editing. **Jean-Yves Madec:** Writing – review & editing, Validation, Resources, Funding acquisition. **Marisa Haenni:** Writing – review & editing, Validation, Project administration, Funding acquisition. **Vincent Perreten:** Writing – review & editing, Conceptualization. **Agnese Lupo:** Writing – review & editing, Supervision, Conceptualization.

## Data availability

Genomes of isolates sequenced in this study are available from the NCBI repository (PRJNA1076118).

## Declaration of generative AI in scientific writing

Authors did not use AI for writing the manuscript.

## Declaration of competing interest

All authors declare no competing interests for this study.
